# Supplementary material for: Dynein–Dynactin–NuMA clusters generate cortical spindle-pulling forces as a multi-arm ensemble
Source: eLife. 2018 May 31;7:e36559. doi: 10.7554/eLife.36559 (PMC6037482; doi:10.7554/eLife.36559)
Supplement: Supplementary file 1. [file elife-36559-supp1.docx]

**Table S1: Cell lines used in this study.**

| **No.** | **Name** | **Description** | **Clone No.** | **sgRNA+template plasmid** | **Parental cell** | **Reference** |
| --- | --- | --- | --- | --- | --- | --- |
| 1 | HCT116 | HCT116 |  |  |  | ATCC CCL-247 |
| 2 | Mem-BFP-iLID | AAVS1::PCMV Mem-BFP-iLID (Puro) | 2 | AAVS1 T2 ( Addgene#72833) and pTK511 | 1 | This study |
| 3 | NuMA-RFP-Nano | AAVS1::PCMV Mem-BFP-iLID (Puro), NuMA1::NuMA-tgRFPt-Nano (Neo) | 4 | pTK372 and pTK421 | 2 | This study |
| 4 | NuMA-RFP-Nano+DHC-SNAP | AAVS1::PCMV Mem-BFP-iLID (Puro), NuMA1::NuMA-tgRFPt-Nano (Neo), DHC1:: DHC-SNAP (Hygro) | 8 | pTK308 and pTK565 | 3 | This study |
| 5 | NuMA-RFP-Nano+p150-SNAP | AAVS1::PCMV Mem-BFP-iLID (Puro), NuMA1::NuMA-tgRFPt-Nano (Neo), DCTN1:: p150-SNAP (Hygro) | 15 | pTK525 and pTK587 | 3 | This study |
| 6 | Nano-mCherry-DHC | AAVS1::PCMV Mem-BFP-iLID (Puro), DHC1::Nano-mCherry-DHC (BSD) | 1 | pTK371 and pTK426 | 2 | This study |
| 7 | Nano-mCherry-DHC+p150-SNAP | AAVS1::PCMV Mem-BFP-iLID (Puro), DHC1::Nano-mCherry-DHC (BSD), DCTN1:: p150-SNAP (Hygro) | 10 | pTK525 and pTK587 | 6 | This study |
| 8 | Nano-mCherry-DHC+NuMA-SNAP | AAVS1::PCMV Mem-BFP-iLID (Puro), DHC1::Nano-mCherry-DHC (BSD), NuMA1::NuMA-SNAP (Hygro) | 8 | pTK372 and pTK583 | 6 | This study |
| 9 | Mem-BFP-iLID+DHC-SNAP | AAVS1::PCMV Mem-BFP-iLID (Puro), DHC1::DHC-SNAP (BSD) | 11 | pTK308 and pTK600 | 2 | This study |
| 10 | DHC-SNAP+RFP-Nano | AAVS1::PCMV Mem-BFP-iLID (Puro), DHC1::DHC-SNAP (BSD), Rosa26:: PTRE3G RFP-Nano (Hygro) | 18 | hROSA26 CRISPR-pX330 (Addgene#105927) and pTK630 | 9 | This study |
| 11 | DHC-SNAP+NuMA(1-2115)-RFP-Nano | AAVS1::PCMV Mem-BFP-iLID (Puro), DHC1::DHC-SNAP (BSD), Rosa26:: PTRE3G NuMA(1-2115)-RFP-Nano (Hygro) | 10 | hROSA26 CRISPR-pX330 and pTK715 | 9 | This study |
| 12 | DHC-SNAP+NuMA(1-2115ΔNLS)-RFP-Nano | AAVS1::PCMV Mem-BFP-iLID (Puro), DHC1::DHC-SNAP (BSD), Rosa26:: PTRE3G NuMA(1-2115ΔNLS)-RFP-Nano (Hygro) | 7 | hROSA26 CRISPR-pX330 and pTK713 | 9 | This study |
| 13 | DHC-SNAP+NuMA(1-1700)-RFP-Nano | AAVS1::PCMV Mem-BFP-iLID (Puro), DHC1::DHC-SNAP (BSD), Rosa26:: PTRE3G NuMA(1-1700)-RFP-Nano (Hygro) | 28 | hROSA26 CRISPR-pX330 and pTK652 | 9 | This study |
| 14 | DHC-SNAP+NuMA(1-705)-RFP-Nano | AAVS1::PCMV Mem-BFP-iLID (Puro), DHC1::DHC-SNAP (BSD), Rosa26:: PTRE3G NuMA((1-705)-RFP-Nano (Hygro) | 5 | hROSA26 CRISPR-pX330 and pTK631 | 9 | This study |
| 15 | DHC-SNAP+NuMA(1-705)4A-RFP-Nano | AAVS1::PCMV Mem-BFP-iLID (Puro), DHC1::DHC-SNAP (BSD), Rosa26:: PTRE3G NuMA(1-705)4A-RFP-Nano (Hygro) | 18 | hROSA26 CRISPR-pX330 and pTK660 | 9 | This study |
| 16 | DHC-SNAP+NuMA(214-705)-RFP-Nano | AAVS1::PCMV Mem-BFP-iLID (Puro), DHC1::DHC-SNAP (BSD), Rosa26:: PTRE3G NuMA(214-705)-RFP-Nano (Hygro) | 2 | hROSA26 CRISPR-pX330 and pTK643 | 9 | This study |
| 17 | DHC-SNAP+NuMA(1-505)-RFP-Nano | AAVS1::PCMV Mem-BFP-iLID (Puro), DHC1::DHC-SNAP (BSD), Rosa26:: PTRE3G NuMA(1-505)-RFP-Nano (Hygro) | 21 | hROSA26 CRISPR-pX330 and pTK664 | 9 | This study |
| 18 | DHC-SNAP+NuMA(1-413)-RFP-Nano | AAVS1::PCMV Mem-BFP-iLID (Puro), DHC1::DHC-SNAP (BSD), Rosa26:: PTRE3G NuMA(1-413)-RFP-Nano (Hygro) | 23 | hROSA26 CRISPR-pX330 and pTK641 | 9 | This study |
| 19 | DHC-SNAP+NuMA(1-213)-RFP-Nano | AAVS1::PCMV Mem-BFP-iLID (Puro), DHC1::DHC-SNAP (BSD), Rosa26:: PTRE3G NuMA(1-213)-RFP-Nano (Hygro) | 8 | hROSA26 CRISPR-pX330 and pTK642 | 9 | This study |
| 20 | DHC-SNAP+NuMA(1-1895)-RFP-Nano | AAVS1::PCMV Mem-BFP-iLID (Puro), DHC1::DHC-SNAP (BSD), Rosa26:: PTRE3G NuMA(1-1895)-RFP-Nano (Hygro) | 4 | hROSA26 CRISPR-pX330 and pTK678 | 9 | This study |
| 21 | DHC-SNAP+NuMA(1-1985)-RFP-Nano | AAVS1::PCMV Mem-BFP-iLID (Puro), DHC1::DHC-SNAP (BSD), Rosa26:: PTRE3G NuMA(1-1985)-RFP-Nano (Hygro) | 19 | hROSA26 CRISPR-pX330 and pTK711 | 9 | This study |
| 22 | DHC-SNAP+NuMA(1-2115Δex24)-RFP-Nano | AAVS1::PCMV Mem-BFP-iLID (Puro), DHC1::DHC-SNAP (BSD), Rosa26:: PTRE3G NuMA(1-2115Δex24)-RFP-Nano (Hygro) | 17 | hROSA26 CRISPR-pX330 and pTK728 | 9 | This study |
| 23 | DHC-SNAP+NuMA(1-705+1700-2115ΔNLS)-RFP-Nano | AAVS1::PCMV Mem-BFP-iLID (Puro), DHC1::DHC-SNAP (BSD), Rosa26:: PTRE3G NuMA(1-705+1700-2115ΔNLS)-RFP-Nano (Hygro) | 4 | hROSA26 CRISPR-pX330 and pTK729 | 9 | This study |
| 24 | DHC-SNAP+NuMA(1700-2115)-RFP-Nano | AAVS1::PCMV Mem-BFP-iLID (Puro), DHC1::DHC-SNAP (BSD), Rosa26:: PTRE3G NuMA(1700-2115)-RFP-Nano (Hygro) | 5 | hROSA26 CRISPR-pX330 and pTK645 | 9 | This study |
| 25 | DHC-SNAP+NuMA(1-2115ΔNLS)5A-3-RFP-Nano | AAVS1::PCMV Mem-BFP-iLID (Puro), DHC1::DHC-SNAP (BSD), Rosa26:: PTRE3G NuMA(1-2115ΔNLS)5A-3-RFP-Nano (Hygro) | 4 | hROSA26 CRISPR-pX330 and pTK746 | 9 | This study |
| 26 | HCT116 tet-OsTIR1 | AAVS1::PTRE3G OsTIR1 (Puro) |  | pAAVS1 T2 and MK243 (Addgene#72835) | 1 | (Natsume et al., 2016) |
| 27 | DHC-mACF | AAVS1::PTRE3G OsTIR1 (Puro), DHC1:: DHC-mAID-mClover-3FLAG (Neo) | 1 | pTK308 and  pTK388 | 26 | This study |
| 28 | NuMA-mACF | AAVS1::PTRE3G OsTIR1 (Puro), NuMA1:: NuMA-mAID-mClover-3FLAG (Neo) | 1 | pTK372 and pTK398 | 26 | This study |
| 29 | NuMA-mACF+DHC-SNAP | AAVS1::PTRE3G OsTIR1 (Puro), NuMA1:: NuMA-mAID-mClover-3FLAG (Neo), DHC1:: DHC-SNAP (BSD) | 18 | pTK308 and pTK600 | 28 | This study |
| 30 | NuMA-mACF+ SNAP-LGN | AAVS1::PTRE3G OsTIR1 (Puro), NuMA1:: NuMA-mAID-mClover-3FLAG (Neo), LGN:: SNAP-LGN (BSD) | 3 | pTK473 and pTK586 | 28 | This study |
| 31 | NuMA-mACF+DHC-SNAP+mCh-NuMA WT | AAVS1::PTRE3G OsTIR1 (Puro), NuMA1:: NuMA-mAID-mClover-3FLAG (Neo), DHC1:: DHC-SNAP (BSD), Rosa26:: PTRE3G mCherry-NuMA(WT) (Hygro) | 7 | hROSA26 CRISPR-pX330 and pTK503 | 29 | This study |
| 32 | NuMA-mACF+DHC-SNAP+mCh-NuMA 5A-3 | AAVS1::PTRE3G OsTIR1 (Puro), NuMA1:: NuMA-mAID-mClover-3FLAG (Neo), DHC1:: DHC-SNAP (BSD), Rosa26:: PTRE3G mCherry-NuMA(5A-3) (Hygro) | 4 | hROSA26 CRISPR-pX330 and pTK750 | 29 | This study |
| 33 | HeLa | HeLa |  |  |  | (Kiyomitsu & Cheeseman, 2012) |
