# Supplementary material for: Dynein–Dynactin–NuMA clusters generate cortical spindle-pulling forces as a multi-arm ensemble
Source: eLife. 2018 May 31;7:e36559. doi: 10.7554/eLife.36559 (PMC6037482; doi:10.7554/eLife.36559)
Supplement: Supplementary file 2. [file elife-36559-supp2.docx]

**Table S2: sgRNA sequences for CRISPR/Cas9-mediated genome editing**

| **Gene locus** | **sgRNA (5'-3')** | **PAM** | **Plasmid Name** |
| --- | --- | --- | --- |
| NuMA1 (C-terminus) | gtggggccactcactggtac | tgg | pTK372 |
| DHC1 (C-terminus) | cctcgcagcttctacgagcg | ggg | pTK308 |
| DHC1 (N-terminus) | cgaggacggctcggccggat | tgg | pTK371 |
| DCTN1 (C-terminus) | agatgaggcgactgtgaagc | tgg | pTK525 |
| LGN | cttataatatgactcgatgg | agg | pTK473 |
| AAVS1 | ggggccactagggacaggat | tgg | AAVS1 T2 ( Addgene#72833) |
| Rosa 26 | ttgcagctcgcgccggtttt | tgg | hROSA26 CRISPR-pX330 (Addgene#105927) |
