# Supplementary material for: Dynein–Dynactin–NuMA clusters generate cortical spindle-pulling forces as a multi-arm ensemble
Source: eLife. 2018 May 31;7:e36559. doi: 10.7554/eLife.36559 (PMC6037482; doi:10.7554/eLife.36559)
Supplement: Supplementary file 3. [file elife-36559-supp3.docx]

**Table S3: PCR primers to confirm gene editing**

| **Gene** | **Primer sequence** | **Primer name** | **Figures** |
| --- | --- | --- | --- |
| AAVS1 | gcattctctcccctgggc | oTK525 | Figure 1-figure supplement 1C |
| Mem-BFP-iLID cassette | gcattcattttatgtttcagg | oCB22 | Figure 1-figure supplement 1C |
| NUMA1 | gagcctcaaagaaggccc | oTK542 | Figure 1-figure supplement 1E  Figure 3-figure supplement 1I Figure 7-figure supplement 1A |
| NUMA1 | agcaggaaccagggcctac | oTK566 | Figure 1-figure supplement 1E Figure 3-figure supplement 1I |
| NUMA1 | ggctgtcccccaacccca | oTK543 | Figure 7-figure supplement 1A |
| DHC1 (C-terminus) | gcgcgcggtaccttcagcgagaggatcaaacagctgc | oTK425 | Figure 1-figure supplement 1F Figure 3-figure supplement 1A Figure 4-figure supplement 1K Figure 7-figure supplement 1A |
| DHC1 (C-terminus) | gcgcgcctcgagtgggccactgtcatcatcaccactg | oTK426 | Figure 1-figure supplement 1F Figure 3-figure supplement 1A Figure 4-figure supplement 1K Figure 7-figure supplement 1A |
| DNCT1 | gccttggtctcagtgctc | oTK658 | Figure 1-figure supplement 1F Figure 3-figure supplement 1I |
| DNCT1 | ggcagaggccagggaatg | oTK659 | Figure 1-figure supplement 1F Figure 3-figure supplement 1I |
| DHC1 (N-terminus) | tgctattcgctgcgtccaca | oTK567 | Figure 3-figure supplement 1H |
| DHC1 (N-terminus) | tctgcagcaccgacacgtcc | oTK568 | Figure 3-figure supplement 1H |
| Rosa 26 | ggtgggaggcgcttgttc | oTK846 | Figure 4-figure supplement 1L Figure 5-figure supplement 1B Figure 7-figure supplement 1A |
| Rosa 26 | aggtgaatgactaagctcc | oTK620 | Figure 5-figure supplement 1B (RFP-Nano) |
| NuMA-RFP-Nano cassette | cgttttgttttgatggagagc | oTK744 | Figure 4-figure supplement 1L Figure 5-figure supplement 1B |
| RFP-Nano cassette | ggcatggacgagctgtacaag | oCB21 | Figure 5-figure supplement 1B (RFP-Nano) |
| mCherry-NuMA cassette | ctgtggggtctgcaggat | oTK445 | Figure 7-figure supplement 1A |
| LGN (N-terminus) | tgtttatagcagaaaatattaca | oTK622 | Figure 7-figure supplement 1A |
| LGN (N-terminus) | ggtccttcagctgaccac | oTK623 | Figure 7-figure supplement 1A |
